# Interspecific and intraspecific Taylor's laws for frog skin microbes

**DOI:** 10.1016/j.csbj.2022.11.061

**Published:** 2022-12-05

**Authors:** Zhidong Liu, Fan Yang, Youhua Chen

**Affiliations:** a China-Croatia “Belt and Road” Joint Laboratory on Biodiversity and Ecosystem Services, Chengdu Institute of Biology, Chinese Academy of Sciences, Chengdu 610041, China; b University of Chinese Academy of Sciences, Beijing 100049, China

**Keywords:** Taylor’s Power Law Extensions, Symbiotic microbial community, Anura microbiota

## Abstract

Amphibians are known to have an abundance of microorganisms colonizing their skin, and these symbionts often protect the host from disease. There are now many comprehensive studies on amphibian skin microbes, but the interspecific and intraspecific abundance distributions (or abundance heterogeneity) of amphibian skin microbes remain unclear. Furthermore, we have a very limited understanding of how the abundance and heterogeneity of microbial communities relate to the body size (or more specifically, skin surface area) of amphibian hosts. In this study, we evaluated the interspecific and intraspecific abundance distribution patterns of amphibian skin microbes and evaluated whether the symbiotic skin microbes of different anuran species share a fundamental heterogeneity scaling parameter. If scaling invariance exists, we hypothesize that a fundamental heterogeneity scaling value also exists. A total of 358 specimens of 10 amphibian host species were collected, and we used Type-I and III Taylor’s power law expansions (TPLE) to assess amphibian skin microbial heterogeneity at the community and mixed-species population levels, respectively. The obtained results showed that, at the community scale, a high aggregation of the microbial abundance distribution on the skin barely changed with host size. In a mixed-species population (i.e., a community context), the abundance distribution pattern of mixed microbial species populations also does not change with host size and always remains highly aggregated. These findings suggest that while amphibian skin microbiomes located in different hosts may have different environmental conditions, they share a fundamental heterogeneity scaling parameter, and thus, scale invariance exists. Finally, we found that microhabitat area provided by the host skin is vital to the stability of the symbiotic microbial community.

## Highlights

• Testing the scale invariance of Taylor’s laws in frog skin microbes.
• Construction of 3D geometric models to measure frog body surface area.
• Host skin space is crucial to the coexistence of the symbiotic microbiota.

## Graphical abstract

**Figure:**
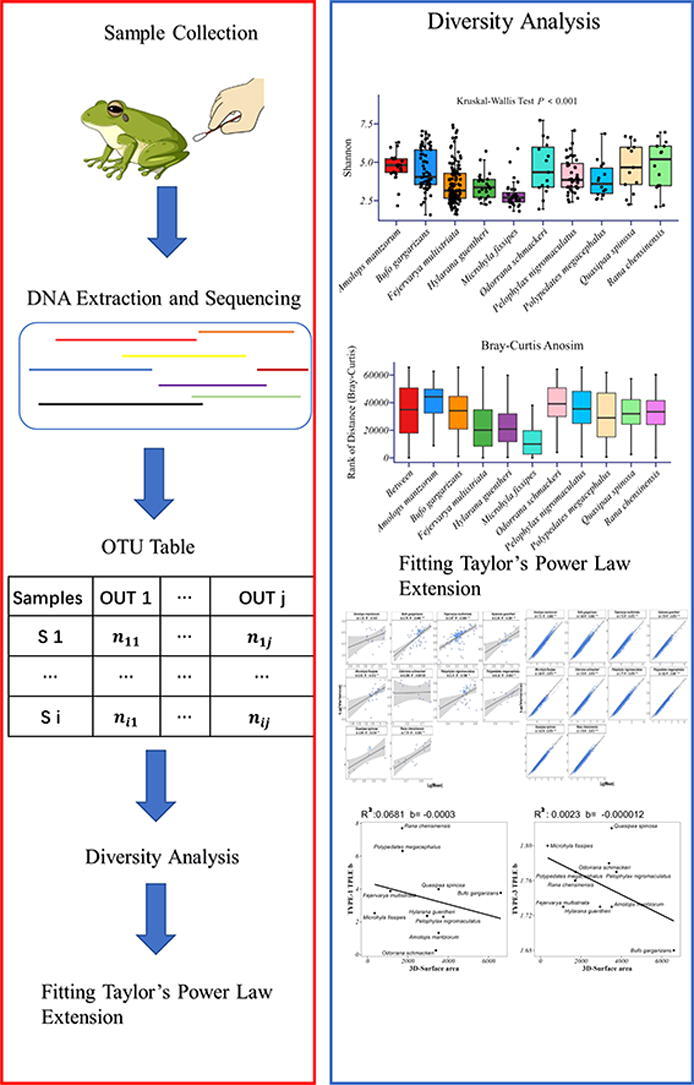

## 1 Introduction

Microorganisms are widespread in every corner of the Earth, and some of them are parasitic on the surface or inside other organisms, playing an important role in maintaining the health of the host and resisting external infestation [1], [2], [3]. With the development of science and technology, especially the emergence of 16S r*RNA* gene sequencing technology, our ability to study microbial communities has been greatly improved. The study of these communities has also improved our understanding of how microbial populations are maintained across generations and the functions of symbiotic microbes. Amphibian skin has an extremely diverse microbial community that can interact with skin cells and influence skin function [4], [5]. Conversely, changes in skin function can also affect the composition of the microbial community [6], [7]. It is an ideal place to study skin-associated microbial communities and provides the best ecological environment for the study of microbial ecology theory [7], [8]. Numerous comprehensive experiments have been performed to study the composition of skin microbiomes in various species [9], [4], but ecological and evolutionary questions of fundamental significance regarding the amphibian skin microbiome remain unclear.

Amphibian skin is one of the most complex organs and is responsible for many physiological functions such as respiration, osmoregulation, excretion, reproduction, thermoregulation and resistance to microorganisms [10], [11], [12], [13]. Amphibian skin contains a mucous layer rich in glycoproteins, which hosts many pathogens and microbial symbionts [14]. At all stages of amphibian life, species-specific oligosaccharides may mediate specific microbial interactions [15], [16]. As a result of lysozymes, antimicrobial peptides, and mucosal antibodies present in the innate and adaptive immune systems, the microbial composition of the skin differs depending on the species [17], [18]. Additionally, amphibians are widespread, and their exposed skin is in direct contact with the environment, making it a vulnerable area for microbial invasion; thus, the skin microbiome composition of individuals living in different regions differs significantly [19], [20]. However, the species abundance distribution pattern of amphibian skin microorganisms has not been directly investigated.

Species abundance and distribution information are indispensable for population and community ecology research. The concept of spatial heterogeneity, which incorporates both species richness and distribution information, has become a representative indicator [21], [22], [23], [24]. In general, amphibian body size and age are positively correlated [25]. If we can anticipate how microbial spatial heterogeneity changes with habitat area, it will help us to study the relationship between the amphibian skin microbial abundance distribution pattern and host age. This is important for studies pertaining to the formation and transformation of skin defense mechanisms during amphibian growth. Taylor's power law [26] is often used in population ecology to quantitatively measure aggregation and heterogeneity, which has been the subject of countless field tests and theoretical analyses since its discovery more than 50 years ago and has become an ecological law in population ecology [27], [28], [29], [30], [31]. Ma extended the original Taylor’s power law into four power law extensions (PLEs), which have become a powerful tool to characterize spatial aggregation (i.e., heterogeneity) of the population and community [32]. Among the four PLEs, Type-I and Type-III PLEs can quantify the community (level) spatial heterogeneity and mixed-species (level) spatial heterogeneity, respectively. TPLE has been applied to microbiome research several times [31], [33], [34], as further explained below.

The objectives of this study were as follows: first, we measured the spatial heterogeneity of amphibian skin microbiomes to assess whether the symbiotic microbial abundance pattern on amphibian skin was random or aggregated. Second, we measured the spatial heterogeneity of the mixed amphibian skin microbial population to assess whether the abundance pattern of symbiotic microorganisms on the skin of different amphibian species was random or aggregated. Third, Ma [32] discovered that whereas hot-spring microbiomes in various parts of the world may have diverse environmental characteristics, they always follow a similar heterogeneity scale parameter [33]. In this study, we wanted to determine whether the symbiotic skin microbes of different amphibians are influenced by external factors and whether a basic heterogeneity ratio parameter also shares a fundamental heterogeneity scaling parameter.

## 2 Materials and methods

### 2.1 Sample collection

From July through August 2020, we collected a total of 358 samples from ten sampling sites around Chengdu, Sichuan Province, China (Figure S1 and Table S1 in the Supporting Information). Prior to sampling each individual animal, all sampling equipment and supplies were disinfected. We ensured protection of the amphibians by only handling the samples while wearing sterile gloves. Before we collected the skin microbes, we rinsed the samples with sterile water to remove any transient bacteria that may have been present on the skin's surface [35]. For uniform sampling standards, we used sterile swabs to rub the head, armpit, back, side, and abdomen of each animal several times to ensure that all skin microbes were collected. We then transferred the swabs to 1.5 ml sterile centrifuge tubes and kept them at −20 °C for *DNA* extraction. All experiments were approved by the Institution of Animal Care and the Ethics Committee of the Chengdu Institute of Biology, Chinese Academy of Sciences.

### 2.2 DNA extraction and sequencing

Following the instructions provided by the manufacturer, the E.Z.N.A. Stool *DNA* Kit (Omega Biotek, Norcross, GA, USA) was used to extract the DNA from the swabs. After *DNA* extraction, we immediately started the PCR process. We amplified the V3-V4 hypervariable regions of the 16S r*RNA* gene using the two universal bacterial primers CCTACGGGNGGCWGCAG and GACTACHVGGGTATCTAATCC. The PCR was carried out in a volume of 30 µl with the following components: 20 ng of the *DNA* template, 10 μM of each primer, 15 μl of 2X KAPA HiFi Hot Start Ready Mix and distilled water. The following were the PCR thermocycling conditions: 1 cycle of denaturing at 95 °C for 3 min, followed by 5 cycles of denaturing at 95 °C for 30 s, annealing at 45 °C for 30 s, and elongation at 72 °C for 30 s, followed by 20 cycles of denaturing at 95 °C for 30 s, annealing at 55 °C for 30 s, elongation at 72 °C for 30 s, and a final extension at 72 °C for 5 min. The PCR amplification products were sent to Sangon Biotech (Shanghai) for high-throughput sequencing on the Illumina MiSeq platform.

After sequencing, we collected data through the following procedures: (1) We assembled Illumina reads using PEAR software (v0.9.6, https://cme.h-its.org/exelixis/web/software/pear/) and processed fastq files to generate individual fasta and qual files for subsequent analysis. (2) We eliminated sequences longer than 480 and shorter than 200 base pairs (bp) and sequences containing ambiguous bases and then allowed homopolymeric sequences with a maximum length of 6 bp [36]. (3) All of the same sequences were merged into one. (4) The alignment of the sequences was performed using a specialized reference database. (5) The index and the adaptor were both checked to ensure that they were complete, and then all of the index and adaptor sequences were removed. (6) The Precluster tool was used to eliminate the noise. Chimera UCHIME (https://mothur.org/wiki/chimera.uchime/) was used to find chimeras. To identify archaeal and bacterial sequences, we resubmitted the effective sequences of each sample to the RDP Classifier. Finally, all sequences with greater than 97 % identity were treated as operational taxonomic units (OTUs) for downstream analysis [37].

### 2.3 Diversity analysis

Statistics were conducted to study the diversity of amphibian skin microorganisms and the differences in the composition of skin microbial communities of different host species. We calculated the variation in alpha and beta diversity among all amphibian skin microbial communities. Here, alpha diversity was measured as the Shannon index. This diversity index is a quantitative indicator of the number of different bacteria that are present in an amphibian skin sample, taking into account the uniformity in the distribution of these bacteria in these species. Beta-diversities of microbial communities were estimated based on Bray–Curtis distances between samples. To determine if there are differences in skin microbial community composition between different host species. A similarity analysis (ANOSIM) was performed using the anosim function in the “vegan” package in R. All statistical analyses were performed using R software, and differences were not significant at P greater than 0.05 but were significant at P ≤ 0.05 and P ≤ 0.01.

### 2.4 Skin area measurement

Different individuals of the same amphibian species have approximately the same skin environment, but there are significant differences in skin area between individuals. To study whether amphibian skin symbiotic microorganisms also have basic heterogeneity scale parameters, we collected the morphological data of the samples, including body length, head width, and head length, and then calculated the skin area of the samples. We used these trait values to construct equivalent three-dimensional geometric models based on the measured morphological trait values. Assuming that head length is denoted as *a*, head width is denoted as *b*, and body length is denoted as *h*, as shown in Fig. S1, we can first construct a cone using the head width as the base diameter and the head length as the height. Then, we constructed a cylinder using the head width as the base diameter and body length as the height. Thus, the three-dimensional skin surface area size (A3) can be computed as:

(1) $$A3=\frac{\pi b\sqrt{a^{2}+\frac{b^{2}}{4}}}{2}+\pi b\left(h-a\right)+\frac{\pi b^{2}}{4}$$

Although we simulated a three-dimensional model using the abovementioned trait values, its accuracy remains to be verified. Therefore, we constructed a two-dimensional model using the abovementioned trait values and conducted an analysis based on 2-dimensional and 3-dimensional data to cross-validate the experimental results and ensure accuracy. As above, head length is denoted as *a*, head width is denoted as *b*, and body length is denoted as *h*. Through two-dimensional geometric transformation (Figure S2), we obtained the two-dimensional skin surface area size (A2) by:

(2) $$A2=\frac{\mathrm{ab}}{2}+b\times \left(h-a\right)$$

Shin area calculation was performed by applying the abovementioned formulae to specimens of different amphibian species (Anura). The A2 and A3 for various species are shown in Table S3.

### 2.5 Analytical methods

Taylor's power law (TPL) was first postulated in 1960 by Lionel Roy Taylor [26], [38], [22], [39], [23] and has since been verified by considerable theoretical and practical studies [40], [41], [42]. The TPL has a mathematical form that describes the relationship between the mean (M) and variance (V) of the biological population in space:

(3) $$V={\mathrm{aM}}^{b}$$

Generally, TPL is fit by transforming Equation (3) into the log-linear model shown below:

(4) $$\ln\left(V\right)=\ln\left(a\right)+bln\left(M\right)$$

where a or $\ln\left(a\right)$ is the intercept, a parameter that, upon examination, is found to be influenced by the sampling effort (sample size) and is of little ecological significance. Parameter b is different from species to species and is usually used to measure the population aggregation (heterogeneity) level, determined by the evolutionary history of the species. The application of TPL has usually been limited to the population level of animals and plants. As mentioned earlier, since TPLE was introduced, it has been widely applied in the study of bacterial communities (see Table S2 for more details). Although TPL and TPLE share the same mathematical form, their interpretations are very different. Type-I PLE can statistically evaluate community spatial heterogeneity,

(5) $$V_{i}={{\mathrm{aM}}_{i}}^{b}i=1,\;2,\cdots ,I$$

where $M_{i}$ is the mean population abundance across all species or operational taxonomic units (OTUs) in the community at sampling site i (i = 1, 2, …,I), $V_{i}$ is the associated variance, and I is the total number of sampling sites.

Similar to the original TPL, a is related to the sampling scheme, but the parameter b reflects the spatial heterogeneity of the community. If the value of b is equal to 1, it means that the community is randomly distributed. If the value of b is greater than 1, it means that the community is highly aggregated. If the value of b is<1, it means that the abundance distribution of all species in the community is equally distributed.

As explained in Table S2, the concept of mixed-species populations refers to populations consisting of the same species (OTU) at several different sampling sites. Type-III PLE was proposed to evaluate the spatial heterogeneity of the mixed-species population abundance if the series of samples is taken spatially [32].

(6) $$V_{j}={{\mathrm{aM}}_{j}}^{b}j=1,\;2,\;\cdots ,J$$

In contrast to the Type-I PLE, $M_{j}$ is the mean of the j-th species (OTU) abundances across all sample sites, $V_{j}$ is the corresponding variance, and j is the number of populations in the mixed-species population (Table S2). If b is equal to 1, it means that the mixed-species population is randomly distributed. If the value of b is<1, the mixed-species population is uniform (regularly) in space. If the value of b is greater than 1, the mixed microbial population is highly aggregated among different sampling sites. Type II and IV PLEs, which quantify the temporal stability of communities, were not implicated in this study.

## 3 Results

**Fig. 1:**
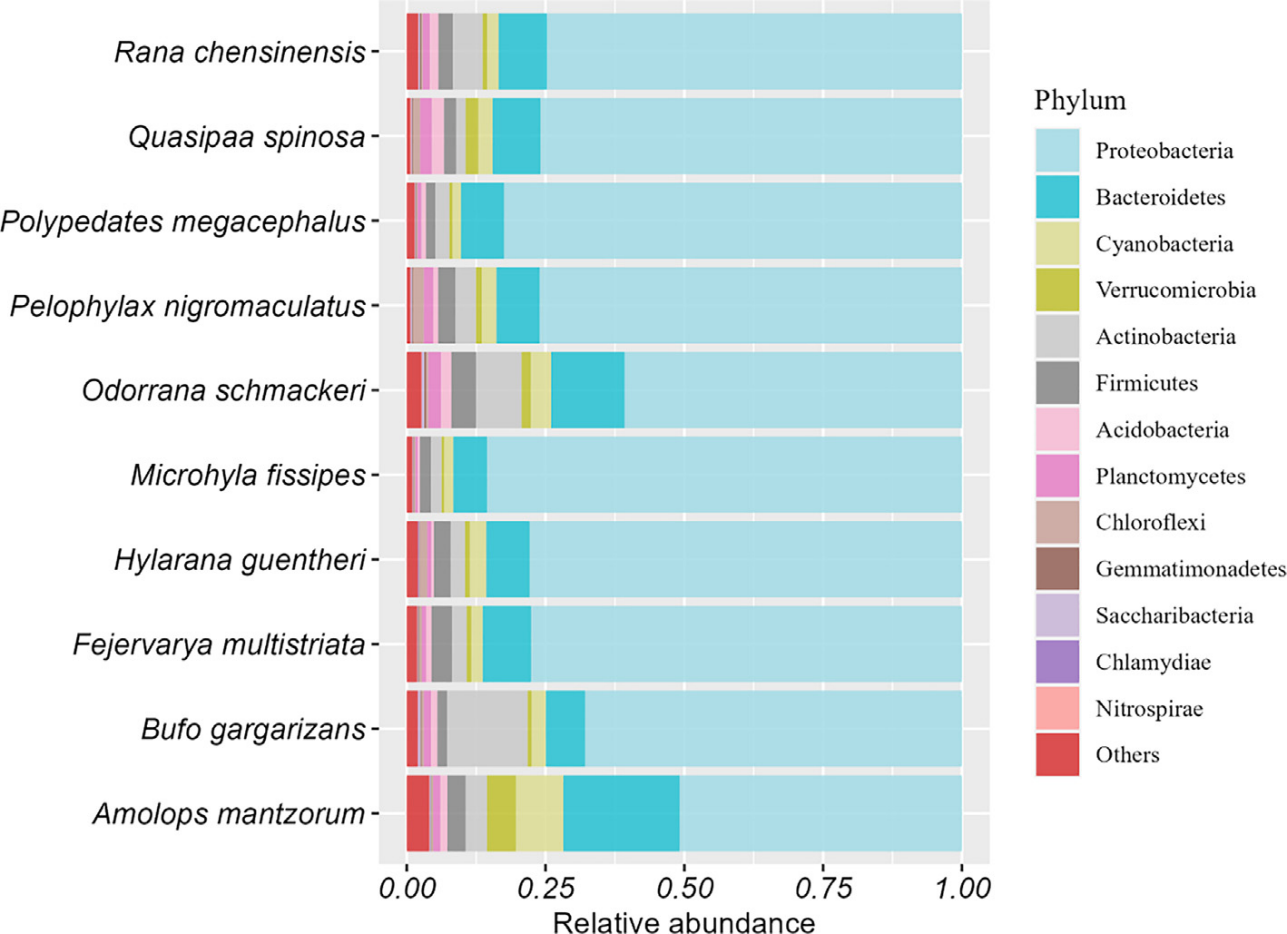
Relative abundance of microbes at the phylum classification level (the top 13 most abundant bacterial phyla) among skin bacterial communities of all amphibian host species studied.

**Fig. 2:**
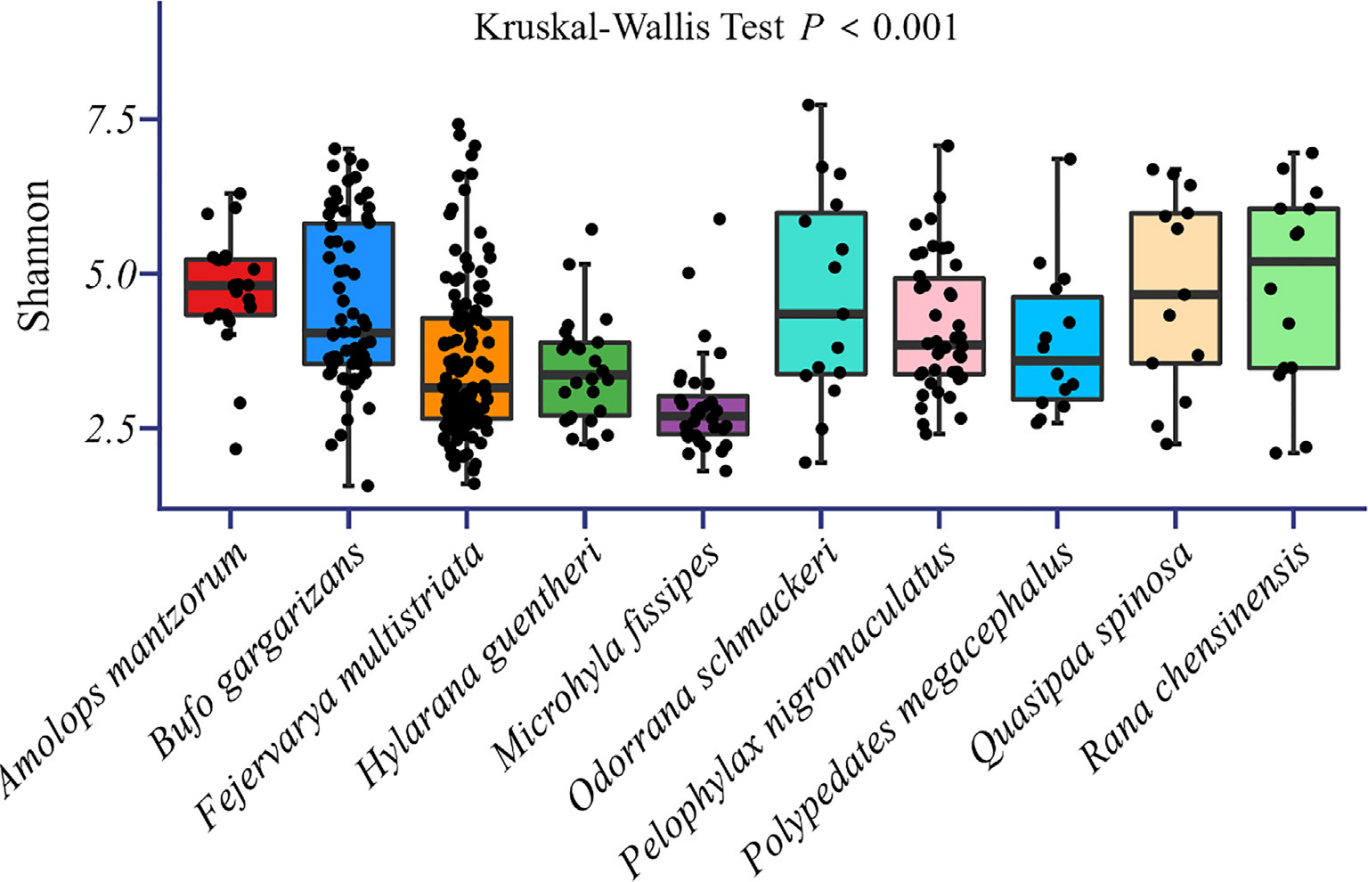
Alpha diversity of different amphibian skin microbial communities. Note that significant differences in the Shannon values were always found between all pairs of different host species (Kruskal-Wallis tests, p < 0.001).

**Fig. 3:**
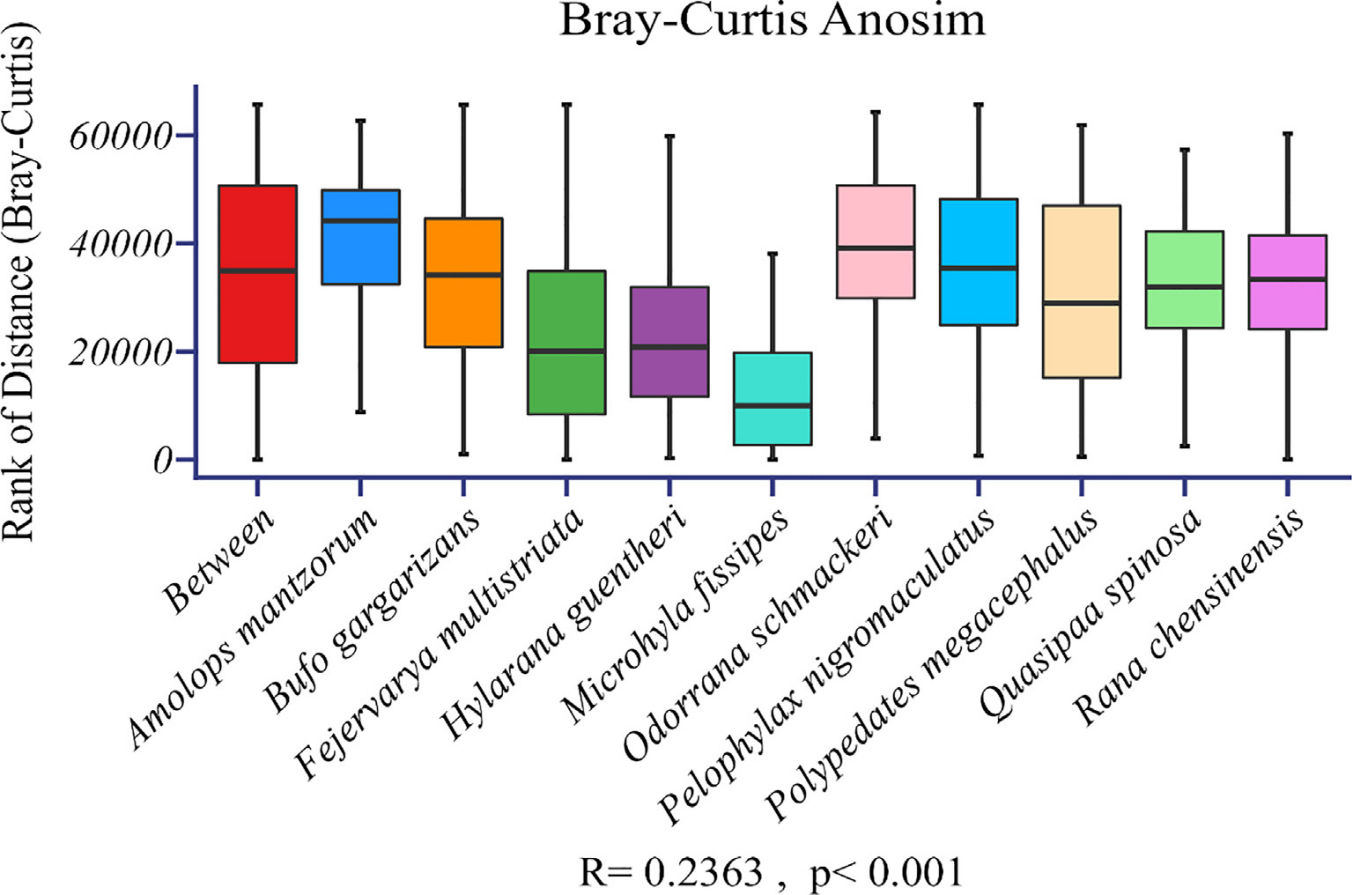
Analysis of similarity (ANOSIM) of the microbial community composition between hosts based on Bray–Curtis distance.

A total of 8837 OTUs were obtained, and the major phyla (i.e., the most abundant ones) were *Proteobacteria* and *Bacteroidetes* (Fig. 1). Our results showed that significant differences in the microbial alpha diversity were found between different host species (Kruskal-Wallis tests, p < 0.001; Fig. 2). Although the dominant skin microbes of different hosts were the same, significant differences in microbial community composition between host species were observed based on the ANOSIM test (Fig. 3 RANOSIM = 0.236, P < 0.001).

**Fig. 4:**
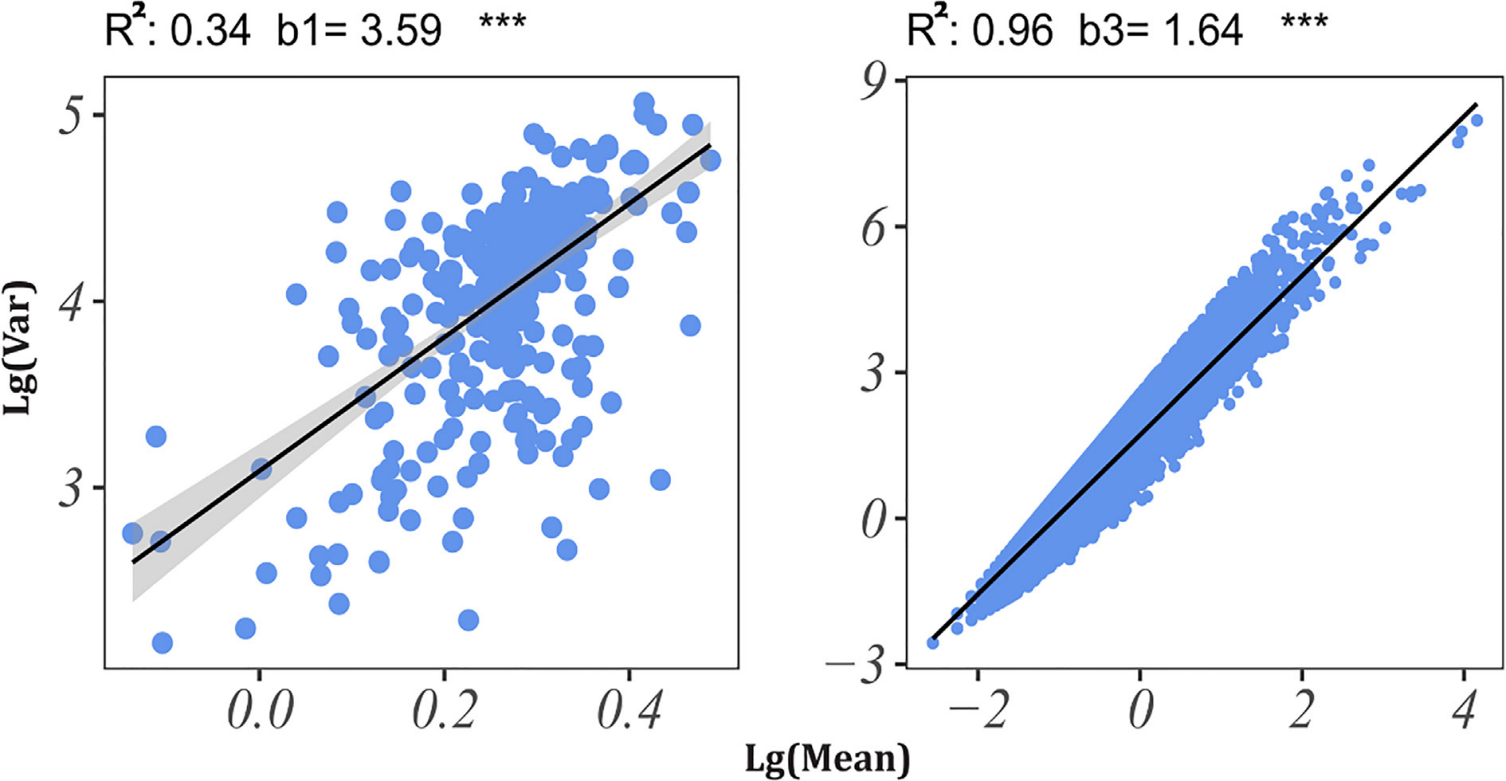
Graph of fitting Type-I PLE (left) and Type-III PLE (right) with all 358 samples of the amphibian skin microbiome. The slope (b1) of the Type-I PLE measures the community spatial heterogeneity; the slope (b3) of the Type-III PLE measures the spatial heterogeneity of mixed species.

To investigate the abundance distribution of amphibian skin microbes, we first fitted the amphibian skin microbiome dataset using Type-I and Type-III PLEs (Fig. 4). The heterogeneity scaling parameter (b1) was much larger than 1, indicating a highly aggregated distribution pattern of amphibious skin microorganisms. Meanwhile, the heterogeneity scaling parameter (b3) was also greater than 1, indicating that the mixed microbial population was also highly aggregated. The mean–variance data points in Type-I PLEs are relatively scattered, indicating that there may be a significant difference in abundance distribution patterns between different host species. In contrast, the mean–variance data points in Type-III PLEs are relatively concentrated, indicating that the abundance of the same microbial is less heterogeneous among different host species.

**Fig. 5:**
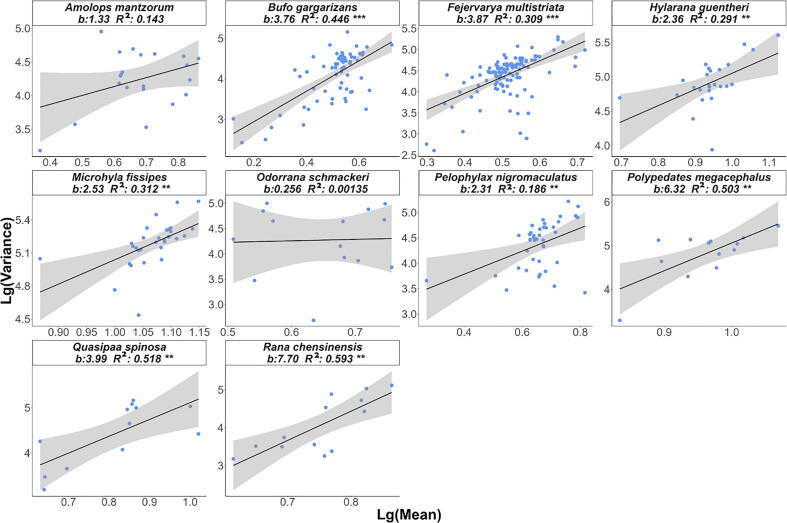
Scatter diagram of the mean variance relationship of symbiotic microbial communities of different host species based on the Type-I model.

**Fig. 6:**
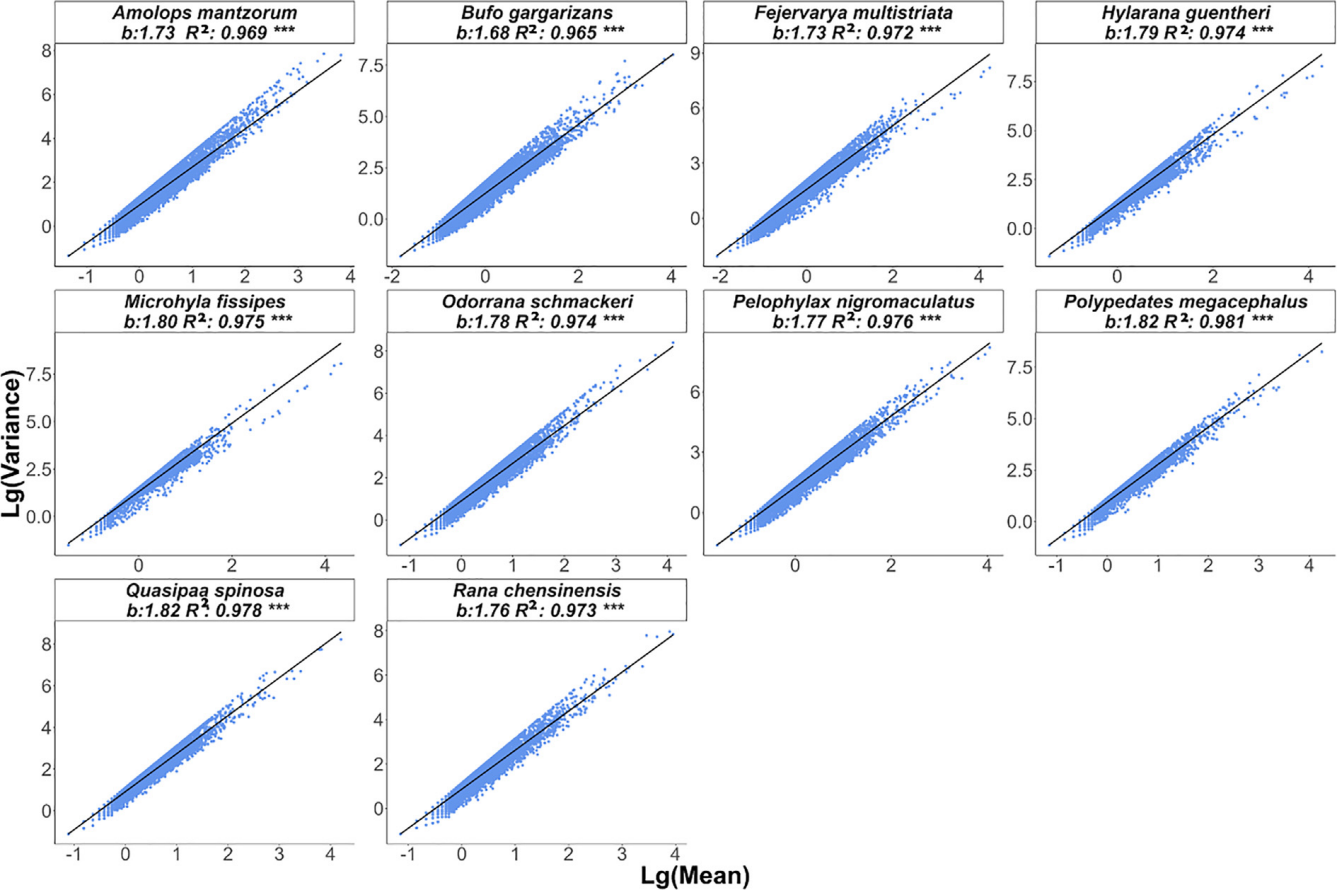
Scatter diagram of the mean variance relationship of symbiotic microbial communities of different host species based on the type III model.

Considering the differences in the composition of skin microbial communities of different amphibians (Fig. 3), it is possible that their distribution patterns also differ significantly. In this case, we fitted Type-I and Type-III PLE models separately to the skin microbial data of each host species. The fitting results are shown in Fig. 5, Fig. 6, and details are shown in Table S3.

The heterogeneity scaling parameters b1 and b3 were different for each species, and they were all greater than 1 except for the Type-I PLE model's slope (b1) of *Odorrana Schmackie*. This means that although the skin microbial communities of different amphibians differ in composition, they all show a highly aggregated distribution pattern. Meanwhile, the mixed microbial population is also highly aggregated among different individuals of the same amphibians. As above, the mean–variance data points in Type-I PLEs are relatively scattered, and the mean–variance ratio of surface microorganisms does not tend to be consistent in different individuals of the same host species, indicating a high heterogeneity in the species abundance of skin microbial communities in different individuals of the same species. In contrast, the mean–variance data points in the Type-III PLE are relatively concentrated, indicating that the abundance of the same microbial communities is less heterogeneous among different individuals of the same host species. However, the parameter b1 value greatly varies among distinct species, which may be caused by the strong biological interaction between symbiotic microorganisms, reflecting the high uncertainty of the changes in the diversity of the microbial community on the body surface. Unlike the Type-I PLE, the heterogeneity scaling parameter (b3) tended to be stable across species.

We can see that the dominant skin microbes are the same across host species (Fig. 1), but do they maintain the same distribution pattern across host species? To answer this question, we selected six dominant phyla to fit the Type-I and III models for validation (Figures S3 and S4). The fitting result shows that the heterogeneity scaling parameters (b1) and (b3) are larger, indicating a more aggregated distribution pattern of dominant microbes on amphibian skin.

**Fig. 7:**
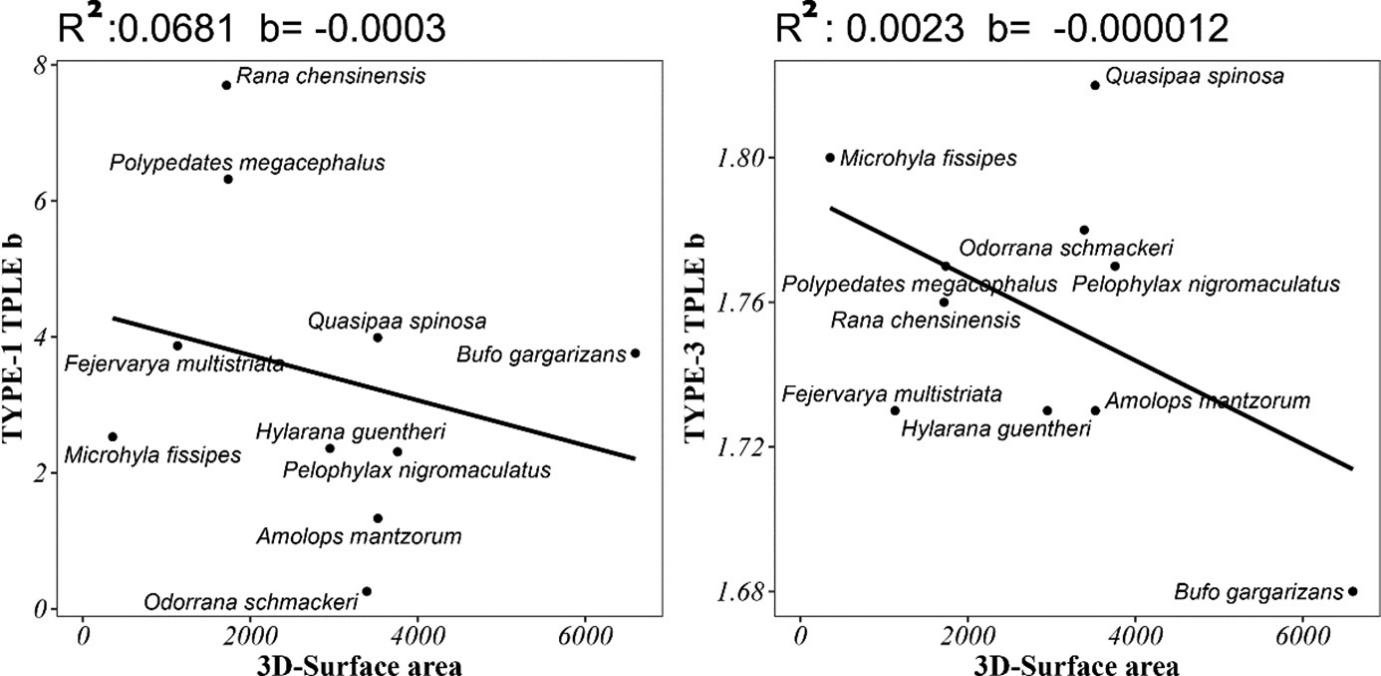
Relationship between the three-dimensional skin area of different host species and parameter b calculated by the corresponding Type-I and Type-III models.

To study whether the skin microbial abundance pattern is influenced by external factors (host body size), we studied the relationship between the heterogeneity scaling parameter (b) and the three-dimensional skin area of the host. The body surface area of each host species is represented by the average of the body skin areas of all individuals of that host species. The obtained results are shown in Fig. 7. The heterogeneity scaling parameters b1 and b3 barely changed with increasing host skin area. This means that the abundance distribution patterns of microbial as well as mixed microbial species populations do not vary with host size and always remain highly aggregated. After cross validation, similar trends are also shown in the heterogeneity scaling parameter (b) and two-dimensional skin area relationship (Figure S5).

Such results are partially inconsistent with the conclusion drawn by a previous study [33]. Ma claimed that in hot spring microbial ecosystems, the Type-I PLE model was not susceptible to microhabitat changes, and there is scale invariance in the heterogeneity scaling parameter b1. Our work showed that frog skin microbes were also not affected by changes in microhabitat area size at different sites and that the b1 value was scale-independent. When studying the mixed-species population distribution pattern, contrary to previous work [33], we found that the heterogeneity scaling parameter b3 was also scale-independent and always remained highly aggregated.

## 4 Discussion

The objectives of this study were as follows. First, we studied the interspecific and intraspecific abundance distributions of amphibian skin microbes. We used the Type-I PLE model to measure the spatial heterogeneity of amphibian skin microbial communities. After rigorous statistical tests on the skin microbes of ten amphibian species, we found that the microbial community was highly aggregated (b = 0.256–7.7). Second, we used the Type-III PLE model to investigate the aggregation of single-microbe populations on amphibian skin. That is, a total of 8837 OTUs were analyzed using a Taylor’s power law model. The results of the study showed that the microbial mixed-species population was also highly aggregated (b = 1.68–1.82). Third, we investigated whether the symbiotic skin microbes of different amphibians are influenced by external factors and share a fundamental heterogeneity scaling parameter. We counted the skin area of different amphibians and investigated the relationship between area and heterogeneity scaling parameter b. The results of the study showed that the heterogeneity scaling parameter (b1 and b3) barely changed with increasing host skin area.

Such result is partially inconsistent with the conclusion reached by Li and Ma [33], who argued that microbial communities in hot springs ought to be heterogeneous. The variance (V) of species abundance is proportional to the mean (m) species abundance on a log scale, and the proportion remains constant (heterogeneity scaling parameter b1) across different temperature and pH regimes. Our results also show that amphibian skin microbial communities are consistently heterogeneous, and the heterogeneity scaling parameter b1 is scale-independent. When studying the hot spring microbial mixed-species population using the Type-III PLE, Li and Ma [33] found that parameter b is influenced by hot spring conditions (temperature and pH). In contrast, our research found that in the amphibian skin microbial mixed-species population context, parameter b3 of the Type-III PLE is not affected by other factors (species and host body size). First, the host spring only provides a suitable living place for microorganisms, and there is no mutually beneficial symbiotic relationship between microorganisms and hot springs. Second, hot springs do not have a strong selective effect on symbiotic microorganisms. In this case, the environment is a strong factor influencing the structure of the microbiome, and the microbial composition considerably varies from one hot spring to another [43], [44]. Most species (OTUs) are present in only a few hot springs, causing a “distortion” of the mean–variance data points; thus, the Type-III parameter (b3), in most groups, exhibited significant differences between different hotspots. In contrast, the relationship between amphibians and skin microorganisms is complex. Amphibians have a selective effect on environmental microorganisms [43], [44], [45], weakening the effect of environmental changes on skin microbial composition. There are no significant differences between skin microbiomes from the same host species, and the vast majority of microbial taxa (OTUs) are present in many hosts. The mean–variance data are calculated by an adequate amount of data and are representative; thus, the Type-III parameter (b3) exhibited no significant differences between different hosts.

We speculate that the reason why amphibian skin microbes exhibit a highly aggregated distribution pattern is because the mucus on the outermost part of the skin is a suitable niche for many microbes and is composed of heavily glycosylated mucins and mucopolysaccharides [46]. Amphibians have various exocrine glands and diverse secretions, providing a variety of ecological niches for microorganism survival. However, the available ecological niche space is far from sufficient, leading to niche differentiation. The wider was the niche breadth, the less pressure there was to promote microbial niche differentiation, and the population abundance showed a less clustered (nondispersed) distribution in space. In contrast, the narrower was the niche breadth, the greater was the pressure of microbial niche differentiation, and the population abundance showed a strong spatial aggregation distribution. The small size of amphibians with narrow niche width leads to a highly aggregated distribution pattern of skin microorganisms. Of note, when studying the spatial heterogeneity of microbial communities, the heterogeneity scale parameter b-values range up to 8, which is quite different from the previous study [33] (0.8 to 2.2). This difference occurs because the main factors affecting the microorganisms living in the hot springs are temperature and pH, and microorganisms in hot-spring environments are highly selective. As such, hot springs in a similar environment are expected to present a similar composition of parasitic microorganisms, and accordingly, the variation in b-values is small. In contrast, there are many factors affecting amphibian skin microorganisms. In addition to temperature and pH, different host species will have significant and differentiated selection effects on microorganisms. Therefore, significant differences in the microbial composition of the skin between host species were observed, leading to much higher variation in the b-value.

Finally, from the microecological perspective, previous studies [47], [48] concluded that the larger is the habitat area, the more stable is the ecosystem. We verified this idea from a microscopic perspective using Taylor's power law. Amphibian skin microbial communities are shaped jointly by a combination of their respective host characteristics and environmental factors. There were significant differences in skin microbial composition across host species. Furthermore, individuals with different body sizes, even for the same species, can also have significantly different skin microbial compositions. This occurs because amphibian body size can influence the effective rate of water loss by affecting surface area-to-volume ratios [49], [50], and differences in water loss rates result in differences in skin properties (a typical example is that the skin humidity of frogs and toads was quite different). Meanwhile, amphibian body size indicates the capacity of physical interactions with the environment and biotic interactions with prey and competitors [51], which in turn affects the skin microbial composition. Although not significant, after comparison, we are able to see that the heterogeneity scale parameter (b1) decreases at a significantly greater rate than b3. This may mean that the larger is the microhabitat provided by the host, the smaller is the variation in microbial abundance and the more stable is the symbiotic community of microorganisms.

Using TPLEs, we were able to assess and compare scaling parameters of amphibian skin microbes at both the community and population levels, which show host species- and microhabitat area-invariant properties. A similar phenomenon occurs in human microbiomes [52], and there are many similarities between these organs. For instance, they all have complex relationships with microorganisms and differ significantly between hosts, and all have a strong selective effect on symbiotic microorganisms. Organisms and their resident microbial communities (i.e., the microbiome) form a complex and mostly stable ecosystem. This may imply that similar phenomena occur in the microbial communities of all similar systems, such as the amphibian guts and fish skins.

## CRediT authorship contribution statement

**Zhidong Liu:** Software, Formal analysis, Writing – original draft, Data curation, Visualization. **Fan Yang:** Writing – original draft, Data curation, Visualization. **Youhua Chen:** Conceptualization, Methodology, Writing – review & editing, Supervision, Project administration, Resources.

## Declaration of Competing Interest

The authors declare that they have no known competing financial interests or personal relationships that could have appeared to influence the work reported in this paper.
