# Supplementary material for: Interspecific and intraspecific Taylor's laws for frog skin microbes
Source: Comput Struct Biotechnol J. 2022 Dec 5;21:251–9. doi: 10.1016/j.csbj.2022.11.061 (PMC9755231; doi:10.1016/j.csbj.2022.11.061)

**Supporting Information**

**Interspecific and intraspecific Taylor's laws for amphibian skin microbes**

Zhidong Liu^1,2#^, Fan Yang^1,2#^,Youhua Chen^1*^

^1^ China-Croatia "Belt and Road" Joint Laboratory on Biodiversity and Ecosystem Services, Chengdu Institute of Biology, Chinese Academy of Sciences, Chengdu, 610041, China

^2^University of Chinese Academy of Sciences, Beijing 100049

#These authors equally contributed to the work

*Corresponding authors: Y.C.; [chenyh@cib.ac.cn](mailto:chenyh@cib.ac.cn)

Table S1. Sampling transect information and number of specimens and species collected in the rural Chengdu region of SW China.

| **transect ID** | **GPS information** | **Host amphibian species recorded** | **Total number of specimens** |
| --- | --- | --- | --- |
| **transect 1** | E.104.2818; N.30.52729 | *Pelophylax nigromaculatus, Quasipaa spinosa, Fejervarya multistriata, Hylarana guentheri, Bufo gargarizans* | 26 |
| **transect 2** | E.104.3245; N.30.41945 | *Pelophylax nigromaculatus, Microhyla fissipes, Fejervarya multistriata, Hylarana guentheri, Bufo gargarizans* | 44 |
| **transect 3** | E.104.0531; N.30.16083 | *Pelophylax nigromaculatus, Microhyla fissipes, Fejervarya multistriata, Hylarana guentheri, Bufo gargarizans* | 36 |
| **transect 4** | E.103.9406; N.29.81665 | *Pelophylax nigromaculatus, Microhyla fissipes, Fejervarya multistriata, Hylarana guentheri, Bufo gargarizans* | 44 |
| **transect 5** | E.103.6009; N.30.02454 | *Pelophylax nigromaculatus, Microhyla fissipes, Fejervarya multistriata, Hylarana guentheri, Rana chensinensis, Bufo gargarizans* | 39 |
| **transect 6** | E.103.5333; N.30.66782 | *Pelophylax nigromaculatus, Quasipaa spinosa, Fejervarya multistriata, Bufo gargarizans, Polypedates megacephalus* | 34 |
| **transect 7** | E.103.3635; N.30.62526 | *Quasipaa spinosa, Fejervarya multistriata, Rana chensinensis, Bufo gargarizans, Odorrana schmackeri, Polypedates megacephalus* | 31 |
| **transect 8** | E.103.2103; N.30.18856 | *Pelophylax nigromaculatus, Microhyla fissipes, Fejervarya multistriata, Rana chensinensis, Bufo gargarizans, Polypedates megacephalus* | 50 |
| **transect 9** | E.103.2596; N.30.18806 | *Bufo gargarizans, Odorrana schmackeri, Amolops mantzorum* | 31 |
| **transect 10** | E.103.2365; N.29.7958 | *Pelophylax nigromaculatus, Fejervarya multistriata, Bufo gargarizans, Polypedates megacephalus, Odorrana schmackeri* | 23 |

Table S2. TYPE I and Ⅲ Taylor’s power law extensions (PLE) at the community level, and the community survey data matrix (i.e.OTU Tables of the amphibian skin microbiome).

| Original Taylor's Power Law | | | $V={aM}^{b}$ | |
| --- | --- | --- | --- | --- |
| Type‐I PLE for Community Spatial heterogeneity | | | ${}^{I}V={a{}^{I}M}^{b}$ | |
| Type­‐III PLE for Mixed-Species Population Spatial heterogeneity | | | ${}^{\mathrm{III}}V=a{{}^{\mathrm{III}}M}^{b}$ | |
| OTU  Sample site | $\mathrm{OTU}_{1}$ | OUT  … | $\mathrm{OTU}_{j}$ | Mean population abundance per species  ,Variance ($M_{j}$,$V_{j}$) |
| Sample 1 | $n_{11}$ | …… | $n_{1j}$ | ${}^{I}{m_{1}}=\frac{1}{j}\sum_{s=1}^{s=j} n_{1s}$  ${}^{I}{v_{1}}=\frac{1}{j}\sum_{s=1}^{s=j} {(n_{1s}-{}^{I}{m_{1}})}^{2}$ |
| …… | …… | …… | …… | …… |
| Sample i | $n_{i1}$ | …… | $n_{ij}$ | ${}^{I}{m_{i}}=\frac{1}{j}\sum_{s=1}^{s=j} n_{is}$  ${}^{I}{v_{i}}=\frac{1}{j}\sum_{s=1}^{s=j} {(n_{is}-{}^{I}{m_{i}})}^{2}$ |
| Mean mixed-species population abundance  ,Variance($M_{i}$,$V_{i}$) | ${}^{\mathrm{III}}{m_{1}}=\frac{1}{i}\sum_{s=1}^{s=i} n_{s1}$  ${}^{\mathrm{III}}{v_{1}}=\frac{1}{i}\sum_{s=1}^{s=i} {(n_{s1}-{}^{\mathrm{III}}{m_{1}})}^{2}$ | …… | ${}^{\mathrm{III}}{m_{1}}=\frac{1}{i}\sum_{s=1}^{s=i} n_{sj}$  ${}^{\mathrm{III}}{v_{1}}=\frac{1}{i}\sum_{s=1}^{s=i} {(n_{sj}-{}^{\mathrm{III}}{m_{j}})}^{2}$ |  |

Table S3. Amphibian skin area and TPLE (Taylor power law extension) fitting results; the body surface area of each species was derived by averaging the body surface area of each individual

|  | body skin area | | Type-I TPLE | | | Type-III TPLE | | |
| --- | --- | --- | --- | --- | --- | --- | --- | --- |
| Host species | A2($\mathrm{cm}^{2}$) | A3($\mathrm{cm}^{2}$) | b1 | $R^{2}$ | b3 | | $R^{2}$ |  |
| *Amolops mantzorum* | 1224 | 3523 | 1.33 | 0.143 | 1.73 | | 0.969 |  |
| *Bufo gargarizans* | 2245 | 6599 | 3.76 | 0.446 | 1.68 | | 0.965 |  |
| *Fejervarya multistriata* | 409 | 1130 | 3.87 | 0.309 | 1.73 | | 0.972 |  |
| *Hylarana guentheri* | 1099 | 2951 | 2.36 | 0.291 | 1.79 | | 0.974 |  |
| *Microhyla fissipes* | 126 | 355 | 2.53 | 0.312 | 1.8 | | 0.975 |  |
| *Odorrana schmackeri* | 1435 | 3391 | 0.256 | 0.0013 | 1.78 | | 0.974 |  |
| *Pelophylax nigromaculatus* | 1353 | 3759 | 2.31 | 0.186 | 1.77 | | 0.976 |  |
| *Polypedates megacephalus* | 621 | 1735 | 6.32 | 0.503 | 1.82 | | 0.981 |  |
| *Quasipaa spinosa* | 1247 | 3521 | 3.99 | 0.518 | 1.82 | | 0.978 |  |
| *Rana chensinensis* | 620 | 1716 | 7.7 | 0.593 | 1.76 | | 0.973 |  |

Figure S1. Sampling site in the rural Chengdu region of SW China.


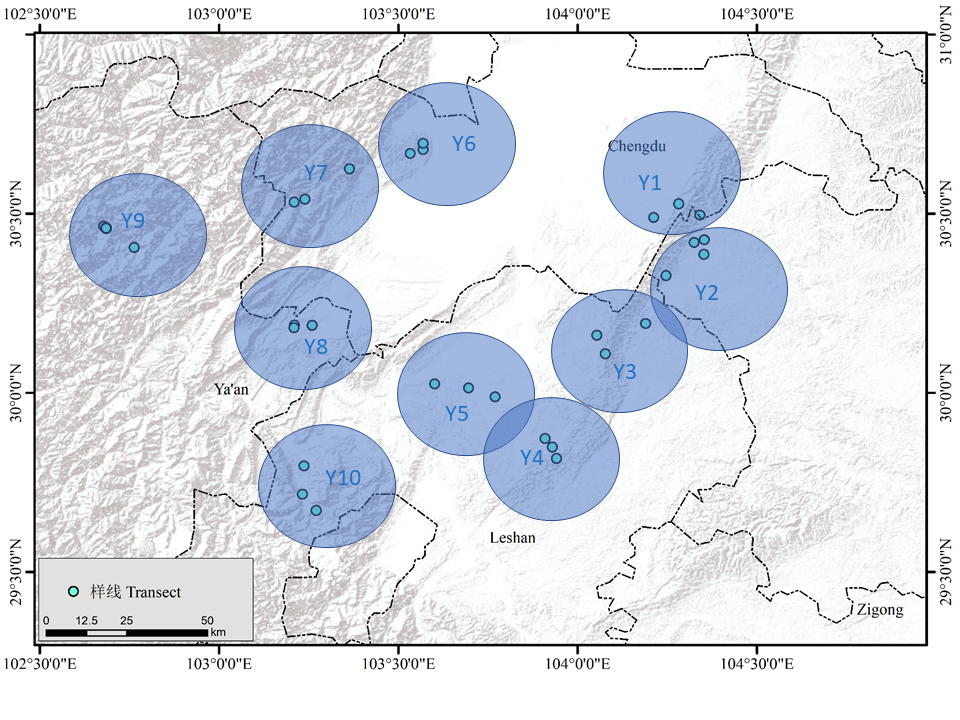


Figure S2. Geometric transformation and calculation of skin microhabitat area size of amphibian hosts. (a-c): Measuring frog body size and equivalent geometric transformations; (d): geometric models for two-dimensional area A2 calculation; (e): geometric models for three-dimensional area A3 calculation.


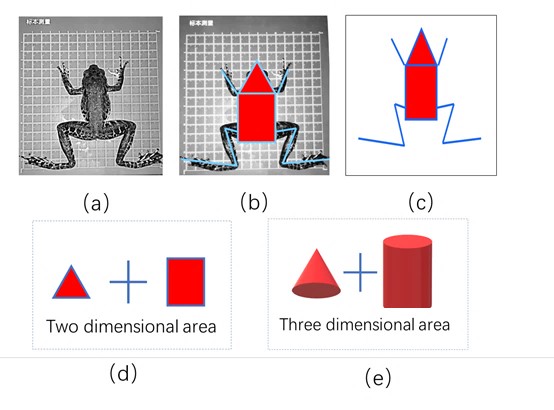


Figure S3. Scatter diagram of mean variance relationship of dominant symbiotic microbial communities of different host-species based on Type-I model
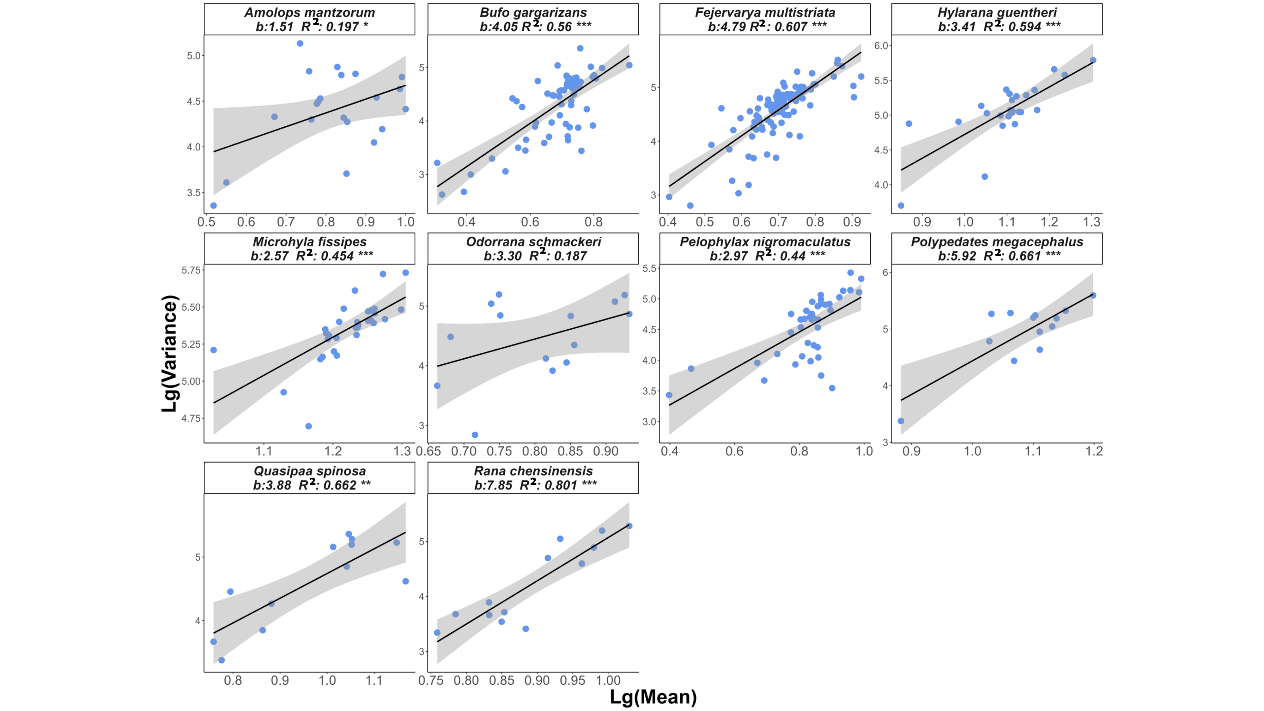


Figure S4. Scatter diagram of mean variance relationship of dominant symbiotic microbial communities of different host-species based on Type-III model


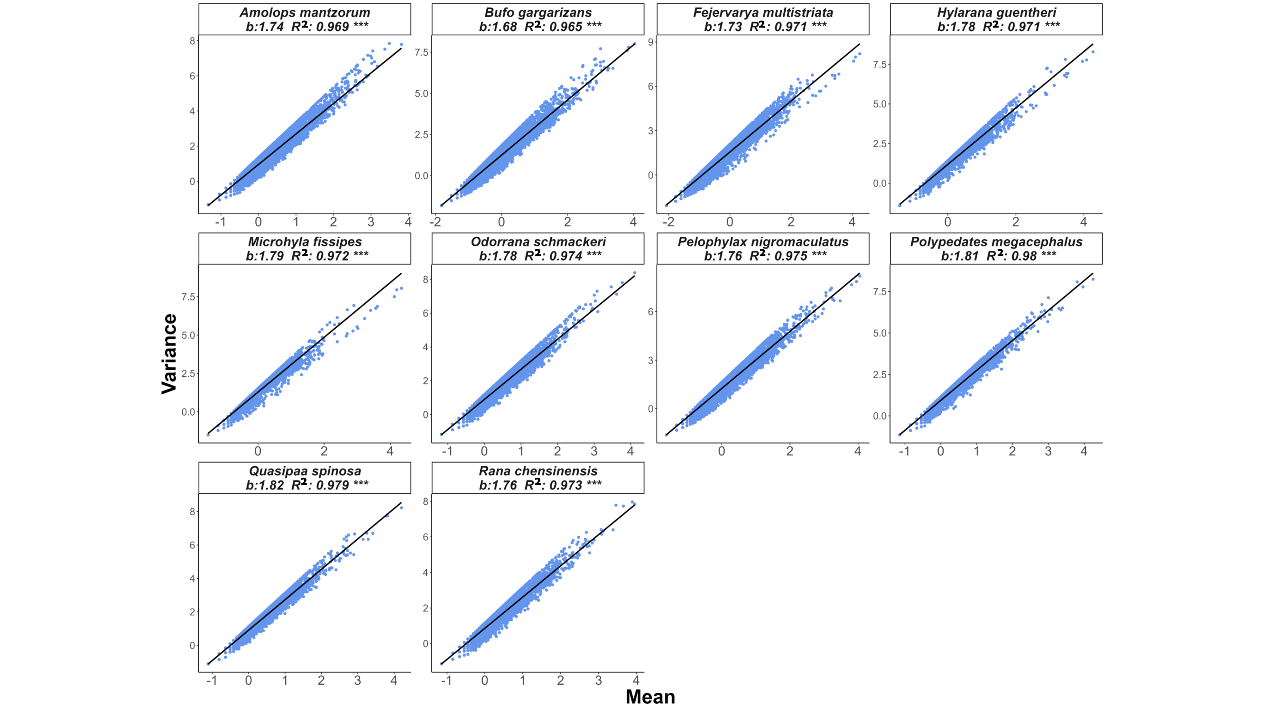


Figure S5. Relationship between the two-dimensional skin area of different host species and the parameter b calculated by the corresponding type-I and type-III models


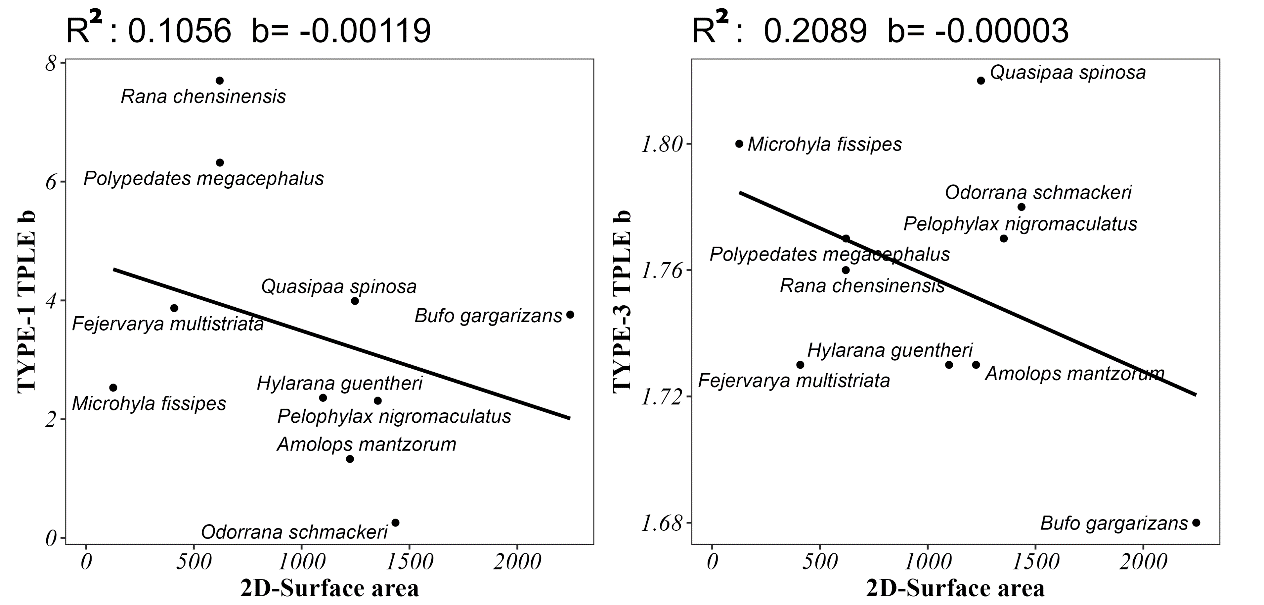

Supplement: Supplementary data 1 [file mmc1.docx]
